# Le syringocystadénome des grandes lèvres: une rare dermatose genital

**DOI:** 10.11604/pamj.2014.18.285.5037

**Published:** 2014-08-10

**Authors:** Douhi Zakia, Mernissi Fatima Zahra

**Affiliations:** 1 Service de Dermatologie-Vénérologie, CHU Hassan II, Fès, Maroc

**Keywords:** Syringocystadénome, grandes lèvres, dermatose génitale, syringocystadenoma:, labia majora, genital dermatosis

## Image en medicine

Les syringocystadénomes papillifères sont des tumeurs annexielles bénignes à différenciation apocrine apparaissant le plus souvent sur le cuir chevelu en association avec un nævus sébacé. Ces lésions peuvent également s'observer isolément dans la région axillaire et inguinale, comme c'est le cas de notre patiente. Ce sont des lésions papuleuses de petite taille, translucides, ombiliquées, parfois suintantes et croûteuses. Le diagnostic clinique est souvent confondu avec un molluscum contagiosum, une verrue, une lésion inflammatoire. A l'histologie, on trouve une lésion kystique profondément invaginée dans le derme et s'abouchant à la surface, tapissée de végétations endoluminales à double assise cellulaire et à différenciation apocrine. Le stroma est riche en plasmocytes. L’évolution de cette lésion est progressive et présentant souvent des poussées inflammatoires et parfois une surinfection. Le traitement est chirurgical. Nous rapportons le cas d'une patiente de 42 ans, ayant reçu il y a 7 ans un traitement à base de radio-chimiothérapie pour un carcinome épidermoide du col utérin, qui consulte pour des lésions asymptomatiques au niveau des grandes lèvres évoluant depuis 3 ans. L'examen clinique révèle la présence de multiples papules translucides et rosés au niveau des grandes lèvres avec une pachydermie pubienne. L'examen dermoscopique a objectivé une zone centrale rouge avec une collerette épidermique blanchâtre périphérique. La patiente a bénéficié d'une biopsie qui a confirmé le diagnostic.

**Figure 1:**
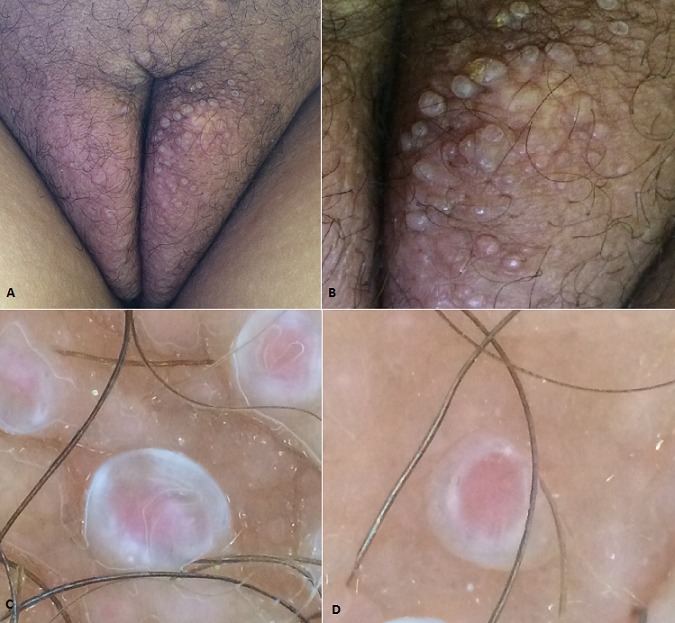
A, B) Multiples papules translucides et rosés au niveau des grandes lèvres, de consistance ferme, indolore avec une pachydermie pubienne post radique; C,D) Aspect dermoscopique des lésions: zone centrale rouge avec une collerette épidermique blanchâtre

